# 

**Published:** 1879-12

**Authors:** 


BOOKS AND PAMPHLETS RECEIVED.

**Physician's Visiting List for 1880.** Lindsay & Blakiston.

**Walsh's Physician's Combined Call-Book and Tablet.** Published by RALPH WALSH, M. D., 326 C. Street, Washington, D. C.

**Walsh's Physician's Handy Ledger,** a companion to Walsh's Physician's Combined Call-Book and Tablet. Published by the same. Price $3.00.

**Transactions of the Medical Society of the State of New York.**

**American Cyclopedia of Domestic Medicine and Household Surgery.** By SAMUEL PAYNE FORD, M. D. E. P. Kingsley & Co., Chicago. 1879.

